# Non-coding RNAs as integrators of the effects of age, genes, and environment on ovarian aging

**DOI:** 10.1038/s41419-019-1334-6

**Published:** 2019-01-28

**Authors:** Danila Cuomo, Concetta Ambrosino

**Affiliations:** 1 Department of Science and Technology, University of Sannio, Via Port’Arsa 11, Benevento, 82100 Italy; 2 Department of Molecular and Cellular Medicine, College of Medicine, Texas A&M University, College Station, TX 77843 USA

One of the significant social changes seen in the past few decades is the decision taken by an increasing number of women, particularly career women, to delay starting a family. These decisions often invite undesirable consequences such as difficulties in conception, and a major contributing factor to such difficulties is ovarian aging^1^. A primary cause of ovarian aging is depletion of the ovarian pool of nongrowing follicles (NGFs)^2^. While this process is a natural consequence of aging, its onset also varies significantly among individuals as indicated by large range in age at which women undergo menopause. It is also becoming increasingly clear that other factors besides normal hormonal cycles influence ovarian aging. In particular, we are now coming to appreciate that large variabilities in diminished ovarian reserve (DOR) between individuals reflect both genetic and environmental factors and, above all, their complex interplay.

Genome-wide association studies have identified at least 44 genomic loci that influence the age of menopause onset^3^. Furthermore, environmental factors (e.g., diet and chemical toxins) also modulate the rate of decline in ovarian reserve (OR). Overall, they can impact processes such as oxidative stress, inflammation and hormone secretion, all contributing to follicular atrophy^4^. Thus, the onset of ovarian aging is recognized as an exceedingly complex process in which age, genetics and environment participate, although the precise mechanisms by which these may do so are poorly understood.

Where do we stand from the perspective of clinical diagnosis of progression of ovarian aging or predisposition to ovarian aging? Unfortunately, there are no genetic or exposure markers for assessing OR available for routine clinical use. Current methods for measuring OR and predicting onset of fertility loss and menopause involve measurements of serum levels of follicle-stimulating hormone, inhibin B, anti-Müllerian hormone, and antral follicle counts. However, none of these parameters accurately estimate the reproductive lifespan of an individual^5^. Thus, new strategies for assessing OR progression, or predisposition are needed. One promising approach to this end is to use gene–environment interaction studies to identify sensitive and specific biomarkers for evaluating the “biological ovarian age”. That is, a reliable measure of ovarian health that does not factor chronological age since ovarian health and chronological age are rather loosely coupled^6^. A productive execution of such an approach requires a detailed understanding of the molecular mechanisms that operate during the ovarian aging process. New diagnostic tools could be rationally designed focusing on the identification of molecules impaired by all the factors influencing ovarian aging.

Such as approach has been undertaken by Cuomo et al.^7^ in their paper recently published in *Cell Death Discovery*. They applied a differential transcriptomics approach in a mouse model system to identify novel candidate biomarkers for reliably estimating ovary lifespan as a function of age, genetic background and exposure to environmental stressors (e.g., diets and endocrine disruptors). The cohort of differentially expressed genes in ovaries from young (3-month-old) as compared to middle-aged (12-month-old) mice highlighted pathways previously shown to be involved in ovarian aging (e.g., EIF2 and mTOR signaling, mitochondrial stress pathways TGF-beta signaling), as well as protein translation systems which had not been previously associated with ovarian aging (e.g., ribosomal proteins, *Rps3*, *Rps24*; regulatory factors, *Eif3a*, *Eif3m*, *Eif4g2*; small nucleolar RNAs, *Snord16a*, *Snora34*).

**Fig. 1:**
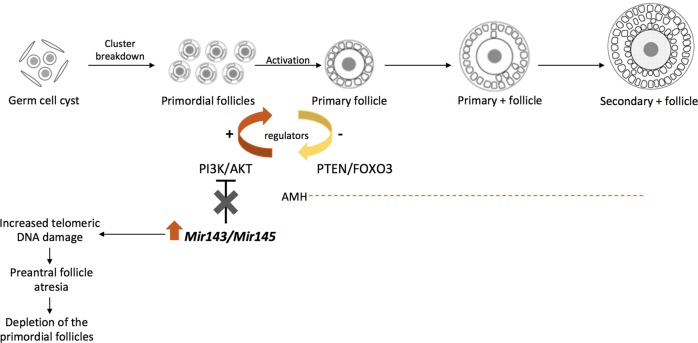
Proposed mechanism linking *Mir143/Mir145* and ovarian aging. Ovarian aging triggers miRNAs dysregulation that would impact on genes/proteins involved in the PI3K/AKT signaling pathway, thereby contributing to the depletion of the primordial follicle pool

The most interesting aspect of the Cuomo et al. study^7^ was the unexpected identification of differentially expressed non-coding RNAs (ncRNAs) in this transcriptomics study. Specifically, the relevant ncRNAs included miRNAs (*Mir143*, *Mir145*, *Mir505*, *Mir681*, and *Mir692-1*), small nucleolar RNAs (*Snord16a* and *Snora34*) and a long non-coding RNA (*Gas5*). Particular attention was focused on *Mir143* and *Mir145*. Indeed, the work of Cuomo et al. represents the first evidence of the association of this microRNA pair with ovarian aging and DOR. The case for these miRNAs as the basis for a diagnostic strategy is reinforced by the demonstration that *Mir143* and *Mir145* expression was consistently deranged by all factors that promote ovarian aging—i.e., age, genetic background, and environmental factors. Moreover, the bioinformatic analyses of the transcripts targeted by both *Mir143* and *Mir145* implicate these miRNAs in regulation of cellular pathways whose compromise is linked to ovarian aging. Specifically, *Mir143* and *Mir145* do have different targets which enrich for common signaling pathways: PI3K/AKT, JAK/STAT, and AMPK. This suggests that miRNAs might act on the same cellular pathways but at different levels and most definitely through different mechanisms. An example is the PI3K/AKT pathway, whose components are known to be required for: (i) follicles development^8^ and (ii) telomere protection^9^ (Fig. 1). Noteworthy, the authors report *Mir681* up regulation in the ovaries from middle-aged mice which is known to functionally suppress AKT^10^. While it remains unclear exactly how these pathways modulate physiological and induced ovarian aging processes, these data nevertheless offer a new path for establishing a mechanistic-based diagnostic marker linking age, genetic, and environmental factors in the ovarian aging process. The fact that *Mir143* and *Mir145* abundance can be followed in follicular fluid (FF) from women is yet another advantage for their development as suitable biomarkers.

How faithfully mouse studies translate to the human condition is always a concern. In that regard, there is no question that much work needs to be done to validate the efficacy of these ncRNA biomarkers in estimating human ovarian aging. But, the early returns are promising. Cuomo and colleagues^7^ present preliminary results showing that FF from women with DOR exhibit elevated *Mir143*, *Mir145* levels. Although those analytical data were derived from a small sample size, the results nonetheless remain consistent with *Mir143* and *Mir145* expression being associated with reductions in mature oocyte loads and compromised oocyte reprogramming capacities (an indicator of MII oocyte quality). The profiling of *Mir143*, *Mir145*, and other ncRNAs in FF samples now offers a promising translational strategy for developing an effective and predictive biomarker platform to monitor ovarian aging in the general human population using noninvasive methods.
